# Is self-weighing an effective tool for weight loss: a systematic literature review and meta-analysis

**DOI:** 10.1186/s12966-015-0267-4

**Published:** 2015-08-21

**Authors:** Claire D. Madigan, Amanda J. Daley, Amanda L. Lewis, Paul Aveyard, Kate Jolly

**Affiliations:** School of Health and Population Sciences, University of Birmingham, Edgbaston, Birmingham B15 2TT UK; School of Social and Community Medicine, University of Bristol, Canynge Hall, 39 Whatley Road, Bristol, BS8 2PS UK; Nuffield Department of Primary Care Health Sciences, University of Oxford, Radcliffe Observatory Quarter, Woodstock Road, Oxford, OX2 6GG UK; The Boden Institute of Obesity, Nutrition, Exercise and Eating Disorders, The University of Sydney, Level 2 Charles Perkin Centre D17, Sydney, NSW 2006 Australia

**Keywords:** Self-weighing, Obesity, Public health, Treatment

## Abstract

**Background:**

There is a need to identify effective behavioural strategies for weight loss. Self-weighing may be one such strategy.

**Purpose:**

To examine the effectiveness of self-weighing for weight loss.

**Methods:**

A systematic review and meta-analysis of randomised controlled trials that included self-weighing as an isolated intervention or as a component within an intervention. We used sub groups to analyse differences in frequency of weighing instruction (daily and weekly) and also whether including accountability affected weight loss.

**Results:**

Only one study examined self-weighing as a single strategy and there was no evidence it was effective (-0.5 kg 95 % CI -1.3 to 0.3). Four trials added self-weighing/self-regulation techniques to multi-component programmes and resulted in a significant difference of -1.7 kg (95 % CI -2.6 to -0.8). Fifteen trials comparing multi-component interventions including self-weighing compared with no intervention or minimal control resulted in a significant mean difference of -3.4 kg (95 % CI -4.2 to -2.6). There was no significant difference in the interventions with weekly or daily weighing. In trials which included accountability there was significantly greater weight loss (p = 0.03).

**Conclusions:**

There is a lack of evidence of whether advising self-weighing without other intervention components is effective. Adding self-weighing to a behavioural weight loss programme may improve weight loss. Behavioural weight loss programmes that include self-weighing are more effective than minimal interventions. Accountability may improve the effectiveness of interventions that include self-weighing.

**Electronic supplementary material:**

The online version of this article (doi:10.1186/s12966-015-0267-4) contains supplementary material, which is available to authorized users.

## Introduction

Finding simple, yet effective, ways in which individuals can be helped to lose weight and sustain weight loss could improve public health. One promising behaviour change technique is to prompt self-monitoring, which has been shown to be an effective technique for healthy eating, physical activity and alcohol reduction [1–3].

Programmes in which participants set a target for their weight, and monitor performance against that target may prove to be an effective stand-alone or adjunct technique for weight loss programmes. Self-weighing is monitoring of the outcome (i.e. weight) rather than behaviour and thus may be used as a prompt to change dietary and physical activity behaviours. There have been two systematic reviews specifically examining self-weighing for weight management and both concluded that regular self-weighing appeared to be a good predictor of moderate weight loss, less weight regain or avoidance of initial weight gain in adults [4, 5]. The first systematic review included a mix of study designs and it was not possible for the authors to do a meta-analysis or identify the key elements of the interventions that might have led to the apparent effectiveness of self-weighing. The second systematic review did not separate the effects of self-weighing for weight loss, prevention of weight regain after weight loss and prevention of weight gain, and there may be differential effects for these interventions. There was also no meta-analysis or estimate of the likely effect of self-weighing. Here we aim to assess self-weighing for weight loss and identify elements associated with greater effectiveness, focusing exclusively on studies with randomised controlled trial designs.

We examine whether self-weighing is effective for weight loss and also examine whether advising people to weigh themselves can be effective as a single intervention or only in the context of a behavioural support programme. If self-weighing can be effective on its own as a prompt to action, then perhaps advice to do so might form the basis of a public health campaign. This is important as many people try and lose weight by themselves rather than seeking advice from a clinician or attending a programme [6]. Having effective techniques people can use for self-regulation of weight is important, as many people would benefit from weight loss. However it could also be recommended by clinicians to help patients manage their weight. If self-weighing can work but only with adjunctive interventions then incorporating advice on self-weighing into behavioural programmes could enhance their effectiveness. Currently, widely used behavioural programmes in the UK advise their participants against weighing themselves. There are also widely expressed concerns that self-weighing may have adverse psychological consequences and we will assess this [7, 8].

We address two theoretical issues. Firstly, for self-weighing to be effective it probably needs to become habitual and this might be easier to achieve if it occurs daily rather than, say, weekly [9]. Daily weighing may also be more effective than weekly because it provides more immediate feedback on how behaviour influences weight and immediate feedback leads to greater learning than feedback that is delayed [10]. Secondly, participants in behavioural weight loss programmes often report that it is the weekly weigh-in that is the most salient component of the programme that keeps them committed to their diet and physical activity plan. This is primarily because it provides accountability as it is done in front of the group leader [11]. We assess here whether accountability enhances the effectiveness of self-weighing. Accountability is defined as creating in a person the sense that someone other than themselves is observing and cares whether they weigh themselves or not.

## Methods

### Trial eligibility criteria

RCTs were included and participants were adults (aged ≥18 years). Trials were included if self-weighing was the main intervention strategy or a strategy within a multi-component intervention. Self-weighing was defined as participants being asked to weigh themselves rather than being weighed as part of a programme. The primary outcome of interest was weight change at programme end defined by the last point of intervention contact. A further outcome was weight change at final follow-up, which in some cases was beyond the end of the intervention. Only trials reported in the English language were included. Trials were excluded if participants were pregnant. Although the initial search was part of a wider search of self-weighing for weight management here, we present only the weight loss trials. A trial was defined as a trial of a weight loss intervention if the aim of the intervention was to achieve weight loss and it enrolled only people of an unhealthy weight. These interventions commonly incorporated strategies for preventing weight regain but the main focus was still on achieving weight loss. Trials were excluded if they enrolled people after weight loss where the prime aim of the intervention was to prevent weight regain or trials that enrolled people that aimed to help prevent gradual weight gain.

### Search strategy

A systematic search of the following databases was conducted: Cochrane central register of controlled trials (CENTRAL, The Cochrane library, CINAHL (EBSCO Host) (1982 to August 2014), MEDLINE (OVID SP) (1946 to August 2014), EMBASE (OVID SP) (1980 to August 2014), PsychInfo (OVID SP) (1806 to August 2014) and Web of Science. ISRCTN and clinical trials registries were also searched. Search terms included: body weight, weight loss, weight maintenance, self-monitoring, self-care, self-weighing and weight monitoring. MESH terms were used where applicable (online Additional file 1). We searched the reference lists of included trials and of three previous systematic reviews of self-weighing and self-monitoring [4, 5, 12].

### Study selection

Two independent reviewers screened all search results (titles and abstracts) for possible inclusion and those selected by either or both authors were subject to full-text assessment. The reviewers were not blinded to trial authors, institution, or publication journal.

### Data collection process

One author independently extracted data using forms based on the Cochrane systematic review data collection forms and a second author checked the forms for any discrepancies [13]. Five authors were contacted for further data and one response was received [14].

### Data items

Information was extracted about the study design, inclusion criteria, participants, study setting, duration of intervention and follow-up, intervention and comparator group weight management strategies, number providing follow-up data, imputation method used for missing weight data and any adverse events. Information was also collected about the two theoretical components proposed to influence the effectiveness of self-weighing; frequency of self-weighing and accountability. We extracted behaviour change techniques based on the CALO-RE behaviour change taxonomy [15] and clustered the techniques to make them more manageable based on previous recommendations [16]. Weight change data for intervention and control groups with standard deviations (SD) were recorded.

### Risk of bias in individual trials

The risk of bias of included trials was assessed in accordance with the Cochrane guidelines [13]. We collected information as detailed in the online Additional file 2. This was independently extracted and checked by another author. A high risk of bias for reporting outcome data was defined as a difference in follow-up rates between the groups of ≥10 % or that there was ≥30 % attrition. Other measures of bias were based on the Cochrane guidelines [13].

### Summary measures

The outcomes of interest were mean weight change from baseline to programme end and weight change from baseline to last follow-up. Follow-up was defined as a period after receiving the last intervention contact and a point of data collection. For each study we extracted weight change for each group reporting the mean, SD of the change, and number of participants contributing data. Where SDs were not presented these were calculated from standard errors.

Studies varied in how they imputed weight change data for those missing follow-up weights. Synthesising such studies’ raw data would create spurious differences due to this. Therefore, we standardised the imputation method by calculating change in weight using baseline weight observed carried forwards (BOCF) [17]. We used BOCF because this mitigates bias that may arise because participants that do less well may be reluctant to be followed up. In one trial [18] weight change was not available but mean baseline and end weight were. The mean weight change and its SD was calculated using a standard formula, which imputes a correlation for the baseline and follow-up weights. This correlation was taken from two previously published trials [19, 20]. One trial used a conservative method of imputation that was similar to this imputation, by adding 0.5 kg to the last weight observed carried forwards. The trial was included within the analysis as presented [21].

### Synthesis of results

Meta-analyses were conducted using Review Manager 5.3. Random effects models were used as the diversity of intervention components and control conditions meant that treatment effects were expected to differ. A pooled mean difference was calculated for weight change at programme end and last follow-up separately and *I*^2^ were reported to quantify heterogeneity. The range of treatment effects from self-weighing was quantified by calculating 95 % prediction intervals providing there were at least four comparisons in a meta-analysis [22]. If there were more than two intervention groups the comparator group was divided by the number of intervention groups and each intervention group was analysed individually.

### Analysis strategy

We examined whether advising self-weighing as a standalone intervention could be effective. We then examined self-weighing as an addition to a behavioural programme in which the same behavioural programme without self-weighing instruction constituted the control group. Within this group, there were two subgroups: trials where self-weighing was the only addition to the behavioural programme and trials where several self-regulatory interventions including self-weighing were added to the behavioural programme. Finally, we examined the largest group of trials in which self-weighing was part of a behavioural intervention that was compared with a minimal or no intervention control group. Within the largest group of trials we used subgroup analysis to examine whether the theoretical propositions we identified were supported by the evidence i.e. daily versus less than daily weighing and accountability. We also conducted a sensitivity analysis to investigate the association of the length of the programme and weight change in this largest group of trials.

## Results

### Study selection

**Fig. 1:**
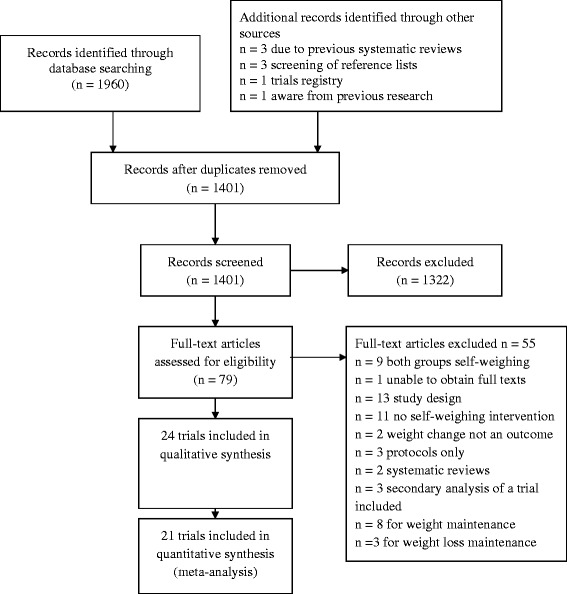
Prisma diagram

Table 1 summarises the participants, interventions, control group intervention and outcome measures that were included within this review. The search identified 1401 studies after duplicates were removed. Titles and abstracts were screened and 79 full text articles were assessed for eligibility. Of those, 24 trials were included in the descriptive synthesis (Fig. 1). The reasons for excluding studies are given in Fig. 1. Data in three trials could only be included descriptively because these studies did not provide standard deviations or data to derive these [23–25].

**Table 1:** PICO for review

PICO	
Participants	Adults – non pregnant.
Interventions	Self-weighing as a standalone or a component of a weight loss intervention.
Control/comparator group	No intervention/comparator or a weight loss intervention that did not include self-weighing.
Outcome	Weight change from baseline to programme end and weight change from baseline to last follow-up point.

### Study characteristics

Table 2 provides a concise summary of the trials and detailed information can be found in the online Additional file 3 and the clustered behaviour change techniques can be found in Additional file 4. All trials were RCTs with the majority conducted in the USA (*n* = 15). The number of participants ranged from 23 to 415 (median 110). Four trials included only women and the percentage of women in the other trials ranged from 26 to 91 % (median 75 %). Eleven interventions used predominantly internet interventions or a mixture of internet and face to face sessions, four were conducted in primary care [26–28]. Intervention length varied from a single session to fifteen months (median: 6 months). Follow-up periods ranged from the end of the intervention to two years. The three most reportedly used clusters of behaviour change techniques were goals and planning, feedback and monitoring, and shaping knowledge (online Additional file 4).

**Table 2:** Brief summary of included trials

Study^a^	Participants n	Duration of intervention	Follow-up	Category^b^	Weighing frequency
Allen et al. (2013 [39]	68	6 months	End of intervention	2	Weekly
Anderson et al. (2014) [32]	329	12 months	End of intervention	3	Weekly
Appel et al. (2011) [26]	415	24 months	End of intervention	3	Weekly
Bacon et al. (2002) [40]	78	6 months	End of intervention	3	Weekly
Batra et al. (2013) Cluster RCT [35]	4 worksites, 118 participants	6 months	End of intervention	3	Daily
Bertz et al. (2012) [34]	68	3 months	End of intervention and 12 months	3	3 times per week
Collins (2012) [29]	309	3 months	End of intervention	3	Weekly
Fujimoto et al. (2002) [30]	72	7.2 months	End of intervention and 24 months	2	4 times daily
Gokee La Rose (2009) [41]	40	10 weeks	End of intervention	2	Daily
Haapalal (2009) [36]	125	12 months	End of intervention	3	Daily
Heckerman et al. (1978) [23]	23	10 weeks	End of intervention and 6 months	3	Weigh often between weekly meetings
Imai et al. (2008) [18]	100	6 months	End of intervention	3	Twice per day
Joachim et al. (1975) [25]	32	8 weeks	End of intervention and 4 months	3	Twice per day
Lally et al. (2008) [43]	104	8 weeks	End of intervention	3	Daily
Leermakers et al. (1998) [42]	90	6 months	End of intervention	3	Weekly
Linde et al. (2011) [31]	68	Single session	6 months	NA	Daily
Ma et al. (2013) [33]	241	15 months	End of intervention	3	Weekly
Madigan et al. (2014) [28]	183	3 months	End of intervention	1	Daily
Mahoney et al. (1973) [24]	53	4 weeks	End of intervention and 4 months	3	Twice per week
Mehring et al. (2013) Cluster RCT [27]	186	3 months	End of intervention	3	Weekly
Pacanowski et al. (2011) [44]	162	12 months	6 months and end of intervention	3	Daily
Steinberg et al. (2013) [37]	91	6 months	End of intervention	3	Daily
Van Wormer et al. (2009) [21]	100	6 months	End of intervention		Weekly
Wing et al. (2010) [38]	128	3 months	End of intervention	2	Daily

^a^All studies are RCTs, unless stated that they are Cluster RCTs

^b^1 = self-weighing isolated, 2 = the same behavioural weight management programme given to both groups but the intervention group were also given self-monitoring/ self-weighing techniques, 3 = self-weighing added to a behavioural weight management programme

### Risk of bias

Risk of bias for individual trials is documented in online Additional file 2. Several trials did not give sufficient information to assess risk of bias in detail. Eleven trials [21, 27–35] were at low risk of bias for sequence generation; for** thef** other trials it was unclear since they did not provide enough information. Seven trials [26–29, 32, 34, 36] had low risk of bias for allocation concealment and four trials were considered as high risk [21, 33, 34, 37],the remainder were unclear.

Two trials [27, 31] did not blind staff to treatment condition at outcome assessment and six trials were classified as low risk of bias for outcome assessment [26, 28, 29, 32, 33, 38], the rest were unclear. All but one trial reported the percentage of participants who were followed up and of these 18 were classified as low and six as high risk of bias [23, 24, 27, 30, 41, 40], because the rate of follow-up differed by more than 20 % between the trial arms or were reported as significantly different. There were only four trials in which selective reporting could be assessed as there was a protocol was available. Three studies were at high risk of bias because they did not report all outcome data [23, 25, 30]. All trials except one used objective data to assess weight change. Fujimoto and colleagues [30] did not report that weight was measured objectively, but follow-ups took place at a hospital so it is probable that weight was measured and not self-reported.

### Synthesis of results

In one study after the initial intervention, participants in both groups were given an optional weight loss maintenance intervention, therefore end of treatment weight only was included in our analysis [40]. Two weight loss trials had a later follow-up and were thus analysed separately [30, 31]. One involved one treatment session and no contact [31] and the other had end of treatment weights and follow-up weights two years from baseline [30]. One trial had more than three intervention groups and a comparator group that received a behavioural weight management programme. We included only the comparator group and the intervention group that received the same programme with additional self-monitoring [39]. Two trials were cluster randomised controlled trials [27, 35]. The trial by Mehring and colleagues did not take account clustering because some clusters included only one participant [27]. The trial by Batra and colleagues [35] did not account for clustering. We undertook sensitivity analysis by removing these two trials from the analysis and in the main outcome, the estimate was reduced by only 0.1 kg and therefore we included them. There was no evidence of subgroup differences in weight change at programme end between programmes that lasted 3 months or less, 6 months, and 12+ months so we analysed all trials together.

**Fig. 2:**
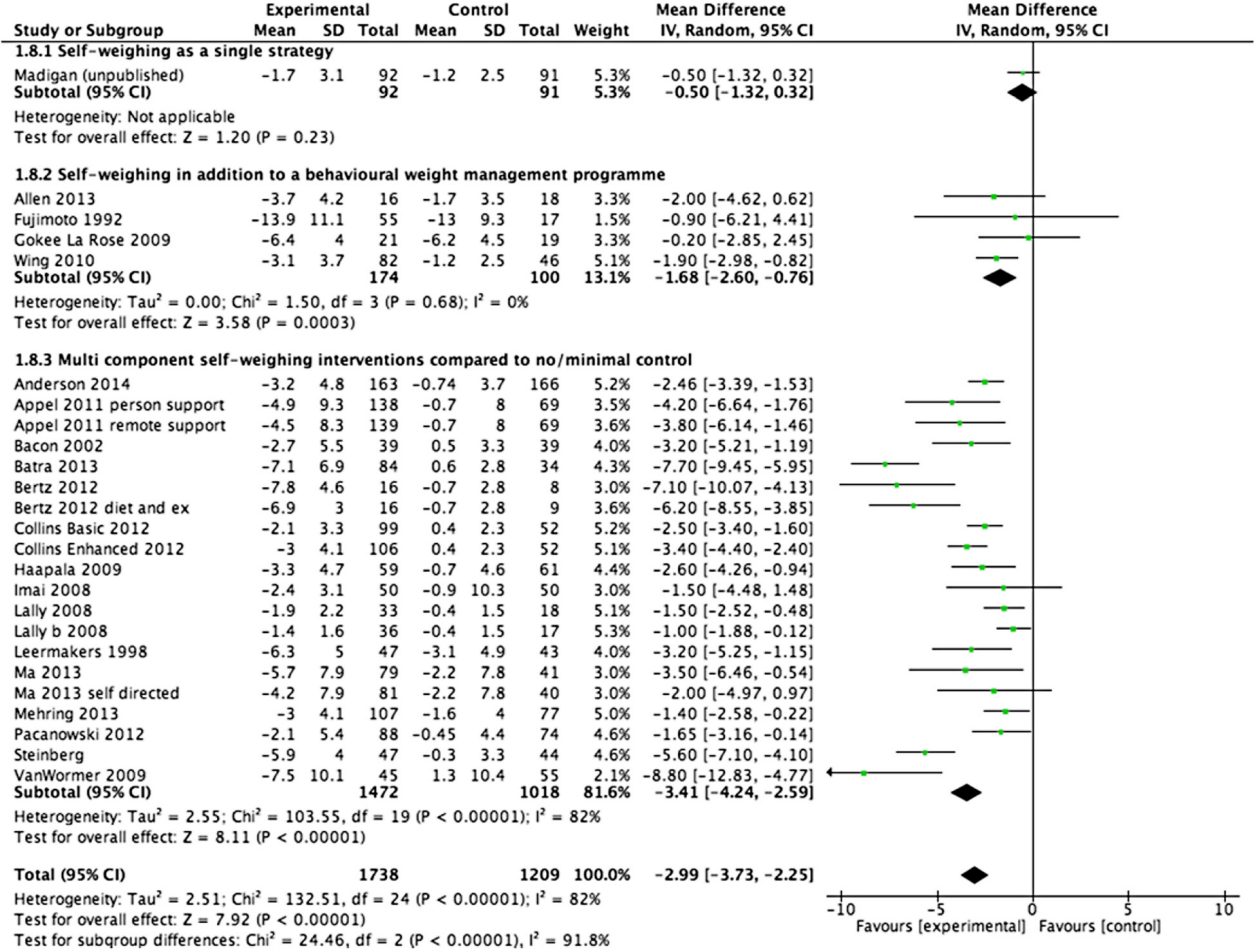
Forest plot of weight loss studies at programme end

A summary of the meta-analyses can be found in Table 3 and Fig. 2 displays the three main groups results in a forest plot. One trial examined the impact of self-weighing without a behavioural programme to achieve weight loss. The mean effect of this intervention was -0.5 kg (95 % CI -1.3 to 0.3 kg) [28]. Four trials [30, 38, 39, 41] compared a behavioural weight management programme plus self-weighing/self-regulation components with a behavioural weight management programme alone. One of these trials included self-regulatory strategies i.e. how to use and interpret the scales like a blood glucose monitor as well as receiving feedback about weight [41]. The other three trials gave participants the option to record their diet and physical activity [38, 39]. The self-weighing/intervention arms had a significantly greater mean weight loss of -1.7 kg (95 % CI -2.6 to -0.8). The prediction intervals ranged from -7.5 to 4.1 indicating that in some interventions participants would lose a considerable amount of weight but in others interventions participants may gain weight. All but one of these trials instructed participants to weigh themselves daily [39].

**Table 3:** Weight change outcomes

		Trials n (number of participants)	Mean difference, kg (95%CI)	*I* ^*2*^	*P*	95 % prediction intervals	Sub group analysis P
Weight change	Mean weight change at programme end	19 (2843)	−3.0 (-3.7 to -2.3)	82 %	<0.01		__
	Mean weight change at follow-up	3 (185)	−5.5 (-11.4 to 4.7)	86 %	0.04	__	__
Self-weighing/self-regulation isolated.	Isolated strategy	1 (183)	−0.5 (-1.3 to 0.3)	__	__	__	__
	Behavioural weight management programme plus self-weighing/self-regulation components compared to the same behavioural programme	4 (274)	−1.7 (-2.6 to -0.8)	0 %	<0.01	−7.5 to 4.1	__
Multi component interventions	All	15 (2490)	−3.4 (-4.2 to -2.6)	82 %	<0.01	−6.9 to 0.1	__
	Daily weighing	7 (795)	−3.6 (-5.4 to -1.8)	91 %	<0.01	−10.2 to 3.0	0.57
	Less than daily weighing	8 (1695)	−3.3 (-4.0 to -2.5)	65 %	<0.01	−4.6 to -1.0	
	Has accountability	14 (2073)^+^	−3.6 (-4.6 to -2.7)	83 %	<0.01	−7.5 to 0.3	0.03
	No accountability	2 (313)^+^	−2.3 (-3.2 to -1.5)	0 %	<0.01	__	

All studies are intention to treat using BOCF ^+^ One trial had three arms and subsequently an intervention arm in each subgroup

Fifteen trials [14, 18, 21, 26, 27, 29, 32–38, 40, 42, 43] were categorised as multicomponent interventions that included self-weighing compared with a no/minimal control group. The mean difference was -3.4 kg (95 % CI -4.2 to -2.6). The 95 % prediction intervals indicate that 95 % of interventions effectiveness would lie between -6.9 to 0.1 kg, indicating most multicomponent interventions including self-weighing would result in weight loss.

#### Theoretical concepts

Of the multicomponent interventions seven trials asked participants to weigh themselves at least daily [18, 21, 35–37, 43, 44]. Eight trials asked participants to weigh less than daily [26, 27, 29, 32–34, 40, 42] and the mean difference was -3.3 kg (95 % CI -4.0 to -2.5). There was no significant difference in the weight differences of the two subgroups (Table 3). Only three trials measured adherence to self-weighing instruction and all three asked participants to weigh daily so we could not examine whether adherence differed between weekly and daily programmes. Adherence was 44 % [36], 50 % [21] and 95 % [37]. A fourth trial instructed participants to weigh daily but asked them to submit weekly logs and found participants did this 76.8 % (SD 23.7 %) of the time [35].

In 14 trials, the intervention group asked to weigh themselves knew that they were accountable to a therapist/researcher [18, 21, 26, 27, 29, 32–37, 40, 42, 43] while this was not the case in two trials [14, 29]. The mean difference between intervention and control groups for those with accountability was -3.6 kg (95 % CI -4.6 to -2.7 kg) and it was -2.3 kg (95 % CI -3.1 to -1.5 kg) for trials without accountability. This difference was significant (p = 0.03). The intervention in two trials had particularly strong accountability because participants knew that the therapist would contact them if they did not weigh themselves [21, 37]. Although there was accountability in other trials, this was more closely related to weight lost and not the act of weighing. The difference between intervention and control groups was larger in the trials with high accountability than in the other trials (-5.6 kg 95 % CI -7.1 to -4.1 kg [37] and -8.8 kg 95 % CI -12.8 to -4.7 kg [21]).

Three trials [30, 31, 34] followed up participants beyond the end of the intervention. The first trial followed up participants approximately 18 months from the last intervention contact and resulted in a mean difference of -8.0 kg (95 % CI -12.5 to -3.5 kg) [30]. The second trial followed up participants six months after the last intervention contact and resulted in a mean difference of -0.3 kg (95 % CI -11.4 to 3.7 kg) [31]. The third trial followed up participants nine months after the last intervention contact and found a mean difference of -7.5 kg (95 % CI -11.3 to -3.7). The three trials that could not be included in the meta analysis found no differences between groups at programme end [23–25].

### Adverse events

Most trials [14, 18, 23–25, 27, 29, 31, 36, 38–40, 42, 43] did not report information about adverse effects.

Three trials measured adverse psychological outcomes by questionnaire. Steinberg and colleagues examined the change in body dissatisfaction, anorectic cognitions, depressive symptoms, dietary restraint, disinhibition, susceptibility to hunger and binge eating episodes between the groups and found no significant differences [45]. Gokee La Rose examined change in depressive symptoms, dietary restraint, body shape concerns, eating concerns, weight concerns and number of binge eating episodes by a trial arm x time interaction [41]. They reported that psychological symptoms improved in both groups and that there were no significant differences in change between groups. In the other trial participants were asked about their mood and how they felt about their body at three months follow-up and there was no difference between groups [28].

Three trials reported there were no serious adverse events related to the intervention in either the self-weighing or control group [21, 26, 32]. One trial detected five serious adverse events possibly related to the intervention but not specifically self-weighing. There were three fractures, one case of chronic subdural hematoma during an intervention session which led to surgery therefore counting twice [33].

Two trials provided non-randomised explanatory analyses to examine further evidence that self-weighing led to adverse psychological outcomes. Steinberg and colleagues conducted a sensitivity analysis of those in the intervention group (instructed to weigh daily) who did not lose weight, and found no difference in body dissatisfaction or depressive symptoms compared to those who did lose weight [45]. This is important, as those who lost weight may have had more positive experiences of self-weighing than those who weighed regularly and didn’t lose weight. Gokee LaRose and colleagues found no relationship between change of frequency of self-weighing and disordered eating [41].

## Discussion

One trial has tested the effectiveness of self-weighing as a single intervention compared with no intervention and there was no evidence that it was effective. There was evidence that adding advice to self-weigh to a behavioural programme improves its effectiveness, but only four trials have assessed this, and the estimate of effect was imprecise and clouded by the use of other self-regulatory elements. There was strong evidence that behavioural weight loss programmes that incorporate self-weighing are more effective than minimal interventions. There was some evidence to suggest that adding accountability to a self-weighing programme improves its effectiveness.

The previous descriptive systematic review of self-weighing using a pre-post analysis found that self-weighing would result in a 5.4 to 8.1 kg weight loss [5]. Our findings are similar, but represent mean differences between intervention and control groups rather than total weight losses and therefore are more conservative and represent the net effect of the self-weighing intervention itself. In the present review only experimental studies with a control group (imputing BOCF for missing weight data) were included which may explain the lower weight change.

Michie and colleagues’ reviews of effective behavioural techniques for healthy eating, physical activity and reduction of alcohol consumption concluded that self-monitoring was effective alone but when combined with other techniques the effect size nearly doubled [1, 2]. The other techniques were prompt intention formation, prompt specific goal setting, prompt review of behavioural goals and provide feedback of performance [1]. However, unlike Michie and colleagues, we found that self-monitoring alone was ineffective for weight loss. However, only one study investigated this and the estimate was imprecise enough to encompass effects that would be worthwhile. Additionally self-weighing is different to the behaviours investigated by Michie and colleagues, as self-weighing is monitoring the outcome rather than the behaviour. To improve the effectiveness of self-weighing additional intervention components may need to be included. This is because people need to reflect on their weight, and then change their dietary and physical activity behaviours. It may be that not all people were prompted to reflect by weighing themselves or were unable to use that reflection to create new strategies to manage their energy intake and expenditure. We did find that adding self-weighing/self-regulation components to a behavioural weight management programme resulted in greater weight loss than the same programme that included no self-monitoring. This suggests that adding self-weighing to a behavioural programme might enhance its effectiveness. Additionally because self-weighing is less cumbersome than recording diet and physical activity it might be a behaviour that can be continued and therefore help weight control in the longer term. The National Weight Control Registry has found that those who are successful at preventing weight regain, after weight loss, weigh themselves on a regular basis [46]. Self-weighing may be used as a strategy to get feedback of cognitive restraint of eating, and this may result in an improved ability to detect changes in weight and thus prompt action if needed.

Multicomponent programmes that included self-weighing compared with a minimal/no intervention comparator group resulted **in significant **weight loss of -3.4 kg (95 % CI -4.2 to -2.6). These findings are similar to a systematic review of behavioural weight management programmes that found a significant difference of -2.6 kg (95 % CI -2.8 to -2.4 kg) [47]. This was 12-18 months after the start of the programme and may explain the smaller mean difference than found when weight loss was assessed at the end of programme only.

We hypothesised that daily self-weighing would more easily lead to the development of habits, however adherence to the self-weighing recommendation was not always reported. There was no evidence that daily weighing led to greater weight loss than weekly weighing and it appears that both may be effective when combined with multi-component interventions. Previous research has examined self-weighing frequency for both weight loss and weight maintenance using a prospective design without a comparison group [48]. Higher weighing frequency was associated with greater weight loss and less weight regain at 24 months follow-up. However, greater motivation to maintain weight or success in achieving weight maintenance may motivate people to weigh themselves frequently, which makes observational data difficult to interpret [49].

We hypothesised that accountability could enhance the effectiveness of self-weighing as participants may feel the need to conform as others were observing what they were doing. Our findings suggested that interventions with accountability had significantly greater weight loss than those without accountability. Gardner and colleagues conducted a systematic review examining similar behaviour change techniques to accountability called audit and feedback [50]. They investigated whether audit and feedback changed healthcare professionals’ behaviour and found a significant effect (OR = 1.43 95 % CI 1.28 to 1.61). Audit and feedback are similar to accountability as participants are aware of being observed, however there is the additional technique of providing feedback which was not necessarily considered within the analyses in our review.

There were no adverse effects of the trials reported, however few trials assessed whether self-weighing led to psychological problems. Those that did, found no evidence of negative consequences.

### Strengths and limitations

This is the first systematic review to include only RCTs to examine the effect of self-weighing. The risk of bias was also reduced by imputing missing weight data using the same method for all studies. There was significant heterogeneity between trials, although this was expected and random effect models and planned sub-group analyses were conducted to investigate this.

Interpreting the data was complicated because there were only a few trials in our sub group analyses and there were differences between trials, such as in length of follow-up, comparator groups, and intervention components. However, we believe that a meta-analysis random effects model is appropriate to investigate whether self-weighing programmes can be effective. Our aim is not to produce a definitive estimate of the effect of self-weighing on weight loss at a particular point in time; rather it is to find evidence that self-weighing as a technique is effective. As length of follow-up is the same in both intervention and control groups, any differences are down either to random variation or differences in effectiveness of self-weighing. Thus the estimates we produce should not be read as estimates of *the* effect of self-weighing, but as valid evidence that self-weighing can be effective in these contexts. Our analyses addressing theoretical constructs were analyses across trials and therefore observational, as no trial directly addressed these issues. We extracted behaviour change techniques used in each intervention, however these were generally poorly reported. It was impossible to separately code those techniques used to promote self-weighing and those that related to other components of the intervention.

### Future research

There was insufficient evidence that self-weighing alone is effective but it is an appealing self-help strategy. Future research should examine other behavioural techniques that can be effectively combined with self-weighing to build low cost public health interventions. Adding accountability may improve the effectiveness of self-weighing. Both daily and weekly weighing may be effective strategies for weight loss but it is not clear whether one is more effective. A trial that is currently being conducted is comparing a behavioural weight management program without self weighing to the same intervention with either daily or weekly weighing [51].

Not all interventions will result in effective weight management for all people and it may prove helpful to identify people who respond to self-weighing and those who do not. Pacanowski reported a subgroup analysis from a trial of self-weighing that found people with internal weight locus of control and males lost more weight [44]. However, indicating that on average some groups respond better does not necessarily make these predictors useful screening tools to exclude people from self-weighing. Given that the advice is apparently simple it may prove that the only screening required is to get people started and react to their responses.

## Conclusions and implications

Self-weighing as part of a multicomponent programme is effective in facilitating weight loss and there is some evidence that indicates adding self-weighing/self-regulation components to a weight loss programme may result in greater weight loss. However as an isolated intervention there is, as yet, no evidence of effectiveness.

## Additional files

Additional file 1:
**Search strategy.** (DOCX 18 kb) Additional file 2:
**Risk of bias.** (DOCX 43 kb) Additional file 3:
**Characteristics of included trials.** (DOCX 46 kb) Additional file 4:
**Number of behaviour change techniques used in each study’s intervention by cluster group.** (DOC 21 kb)

## Abbreviations

BMI: Body mass index
BOCF: Baseline weight observed carried forward
CI: Confidence interval
RCT: Randomised controlled trial
SD: Standard deviation
UK: United Kingdom
USA: United States of America
