# Erratum to: Is self-weighing an effective tool for weight loss: a systematic literature review and meta-analysis

**DOI:** 10.1186/s12966-016-0366-x

**Published:** 2016-03-30

**Authors:** Claire D. Madigan, Amanda J. Daley, Amanda L. Lewis, Paul Aveyard, Kate Jolly

**Affiliations:** School of Health and Population Sciences, University of Birmingham, Edgbaston, Birmingham B15 2TT UK; School of Social and Community Medicine, University of Bristol, Canynge Hall, 39 Whatley Road, Bristol, BS8 2PS UK; Nuffield Department of Primary Care Health Sciences, University of Oxford, Radcliffe Observatory Quarter, Woodstock Road, Oxford, OX2 6GG UK; The Boden Institute of Obesity, Nutrition, Exercise and Eating Disorders, The University of Sydney, Level 2 Charles Perkin Centre D17, Sydney, NSW 2006 Australia

## Erratum

Since publication of the original article [1], a reader observed an error in one of the studies that had been included. The study by VanWormer et al [2] presented the results as pounds and was mistakenly analysed in kg. The results have been re-analysed.

Comparing multi-component interventions including self-weighing with no intervention or minimal control is changed by 0.1 kg (3.3 kg, 95 % CI -4.1 to -2.8). The 95 % prediction intervals changed slightly (-6.7 to 0.05 kg versus previously -6.9 to 0.1). Figure 2 of the original article should have presented these results, as appears correctly within this erratum.

**Fig. 2:**
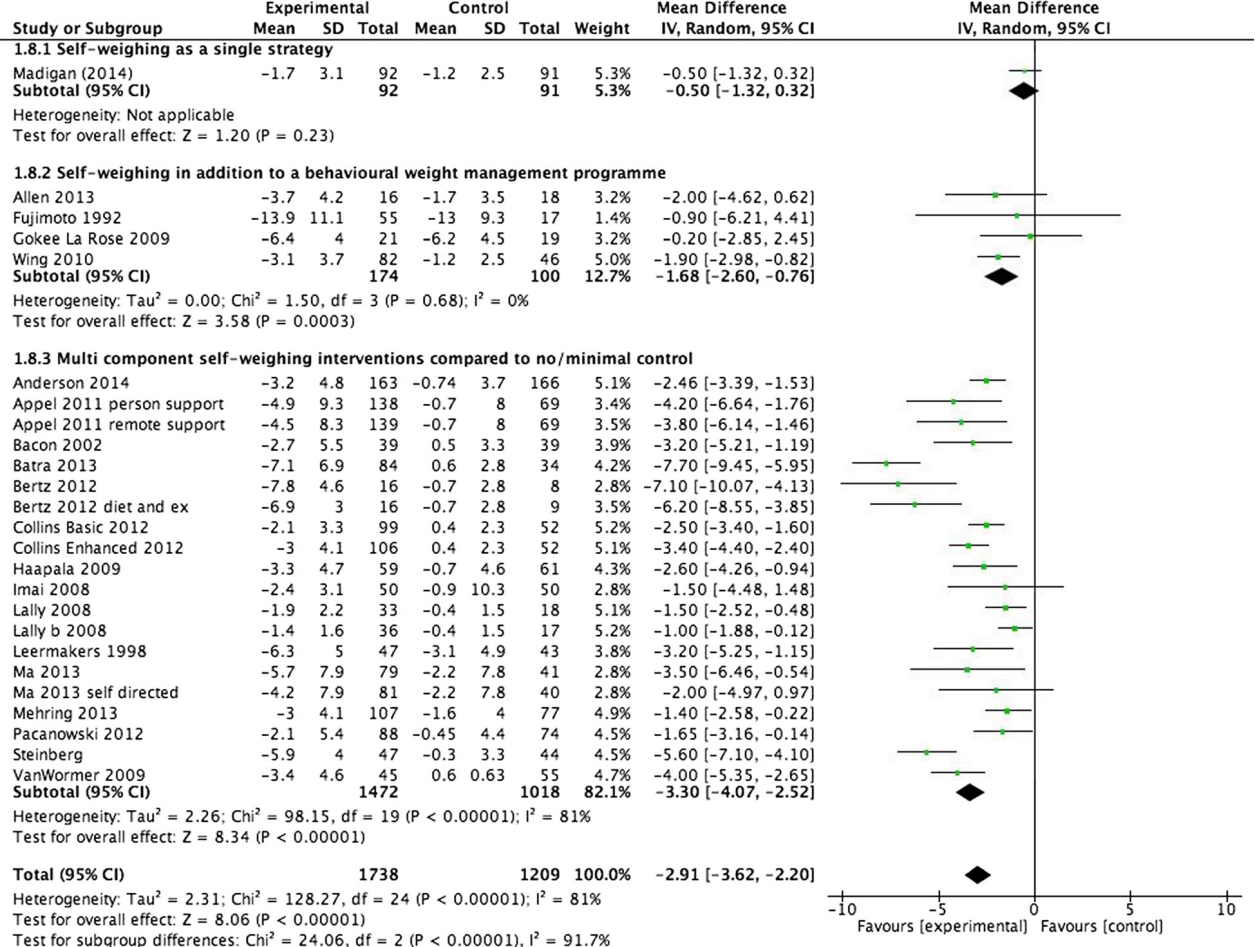
Forest plot of weight loss studies at programme end

In addition, the mean difference between intervention and control groups for those with accountability changed from -3.6 kg (95 % CI -4.6 to -2.7 kg) to -3.5 kg (95 % CI -4.4 to -2.6 kg). This difference was approaching significance (p = 0.05) rather than previously being significant (p = 0.03). An amended version of Table 3 appears here to highlight these changes.

**Table 3:** Weight change outcomes

		Trials n (number of participants)	Mean difference, kg (95 % CI)	*I* ^*2*^	P	95 % prediction intervals	Sub group analysis P
	Weight Loss						
Weight change	Mean weight change at programme end	20 (2947)	-2.91(-3.6 to -2.2)	81 %	<0.01		__
	Mean weight change at follow-up	3 (185)	-5.5 (-11.4 to 4.7)	86 %	0.04	__	__
Self-weighing/self-regulation isolated.	Isolated strategy	1 (183)	-0.5 (-1.3 to 0.3)	__	__	__	__
	Behavioural weight management programme plus self-weighing/self-regulation components compared to the same behavioural programme	4 (274)	-1.7 (-2.6 to -0.8)	0 %	<0.01	-7.5 to 4.1	__
Multi component interventions	All	15 (2490)	-3.3 (-4.1to -2.8)	81 %	<0.01	-6.7to 0.05	__
	Daily weighing	7 (795)	-3.2 (-4.8 to -1.6)	90 %	<0.01	-9.5 to 3.1	0.95
	Less than daily weighing	8 (1695)	-3.3 (-4.0 to -2.5)	65 %	<0.01	-4.6 to -1.0	
	Has accountability	14 (2177)^+^	-3.5 (-4.4 to -2.6)	82 %	<0.01	-8.9 to 1.9	0.05
	No accountability	2 (313)^+^	-2.3 (-3.2 to -1.5)	0 %	<0.01	__	

All studies are intention to treat using BOCF + One trial had three arms and subsequently an intervention arm in each subgroup

## References

[1] Madigan CD, Daley A, Lewis A, Aveyard P, Jolly K (2015). Is self-weighing an effective tool for weight loss: a systematic literature review and meta-analysis. Int J Behav Nutr Phys Act.

[2] VanWormer JJ, Martinez AM, Benson GA, Crain AL, Martinson BC, Cosentino DL (2009). Telephone counseling and home telemonitoring: the weigh by day trial. Am J Health Behav.

