# Supplementary material for: Is self-weighing an effective tool for weight loss: a systematic literature review and meta-analysis
Source: Int J Behav Nutr Phys Act. 2015 Aug 21;12:104. doi: 10.1186/s12966-015-0267-4 (PMC4546162; doi:10.1186/s12966-015-0267-4)
Supplement: Additional file 1: — Search strategy. (DOCX 18 kb) [file 12966_2015_267_MOESM1_ESM.docx]

**Online Additional file 1: Search strategy**

| 1 | Body Weight/ (MESH term) |
| --- | --- |
| 2 | limit 1 to (english language and humans) |
| 3 | self-monitoring.mp. |
| 4 | limit 3 to (english language and humans) |
| 5 | Self Care/ (MESH term) |
| 6 | limit 5 to (english language and humans) |
| 7 | 2 and 3 |
| 8 | 2 and 6 |
| 9 | Weight Loss/ (MESH term) |
| 10 | limit 9 to (english language and humans) |
| 11 | 6 and 10 |
| 12 | 4 and 10 |
| 13 | weight maintenance.mp. |
| 14 | limit 13 to (english language and humans) |
| 15 | 6 and 14 |
| 16 | 4 and 14 |
| 17 | 11 or 12 or 15 or 16 |
| 18 | self-weighing.mp. |
| 19 | limit 18 to (english language and humans) |
| 20 | weight monitoring.mp. |
| 21 | limit 20 to (english language and humans) |
| 22 | 17 or 19 or 21 |
