# Supplementary material for: Is self-weighing an effective tool for weight loss: a systematic literature review and meta-analysis
Source: Int J Behav Nutr Phys Act. 2015 Aug 21;12:104. doi: 10.1186/s12966-015-0267-4 (PMC4546162; doi:10.1186/s12966-015-0267-4)
Supplement: Additional file 2: — Risk of bias. (DOCX 43 kb) [file 12966_2015_267_MOESM2_ESM.docx]

**Online Additional file 2: Risk of bias**

| **Study** | **Sequence of generation** | **Allocation concealment** | **Blinding of outcome assessment** | **Incomplete outcome data** | **Selective reporting** | **Other bias**  **Measurement of weight outcome** |
| --- | --- | --- | --- | --- | --- | --- |
| Allen et al [[38](#_ENREF_38)] | Unclear risk of bias: randomisation procedure not stated | Unclear risk of bias: allocation concealment not stated. | Unclear risk of bias: not stated. | High risk of bias: 63% of participants were followed up at six months. No method of imputation | Unclear risk of bias | Low risk of bias: weight measured. |
| Anderson et al [[31](#_ENREF_31)] | Low risk of bias: participants were randomised 1:1 by an list prepared by an independent statistician using a permuted block technique with block size of four and eight stratified by trial site. | Low risk of bias: research nurses allocated participants a site specific identification number and notified study administrator who then identified the groups allocation number from the randomisation list and notified the lifestyle counsellor. | Low risk of bias: the study team were blinded to the participants group allocation until completion after the primary outcome analysis. | Low risk of bias: 93% followed up at 12 months. Used multiple imputations for missing values. | Low risk of bias: published protocol and reported outcomes. | Low risk of bias: weight measured. |
| Appel et al[[25](#_ENREF_25)] | Low risk of bias: Randomisation was stratified and generated in blocks of three and six using a web based programme. | Low risk of bias: Used a web based programme. | Low risk of bias: The research staff who notified participants of assignment were not involved in collection of follow-up data. | Low risk of bias: 94.5% follow-up rates and groups had similar follow-up rates. Imputed missing weight data using random sequence. | Unclear risk of bias | Low risk of bias: weight measured. |
| Bacon et al[[39](#_ENREF_39)] | Unclear risk of bias: “Participants were divided into BMI quartiles, age, high/ low sets for dietary restraint, degrees of flexible and rigid control of eating, and self-reported activity level and then randomised.” | Unclear risk of bias: allocation concealment not stated. | Unclear risk of bias: not stated. | High risk of bias: At 24 weeks 59% of IG and 92% of the CG provided data. No method of imputation. | Unclear risk of bias | Low risk of bias: weight measured. |
| Batra et al [[34](#_ENREF_34)] | Low risk of bias: one number was assigned to each worksite and a random order of the numbers was generated. The first two worksites were assigned to the intervention. | Unclear risk of bias: allocation concealment not stated. | Unclear risk of bias: not stated | Low risk of bias: high follow up rates and no difference between the groups. Only used intention to treat analysis. | Unclear risk of bias | Low risk of bias: weight measured. |
| Bertz et al [[33](#_ENREF_33)] | Low risk of bias: women were stratified on the basis of pre-pregnancy BMI and a blocked randomisation was used within each stratum. | Low risk of bias: group allocation was concealed until completion of baseline measurements. | Unclear risk of bias: not stated. | Low risk of bias: 91% were followed up at programme end and 84% at one year follow up. | Unclear risk of bias | Low risk of bias: weight measured. |
| Collins et al[[28](#_ENREF_28)] | Low risk of bias: Stratified randomisation block design with variable block lengths of 3 or 6 generated by a statistician. | Low risk of bias: A researcher not involved in data collection distributed sequentially numbered sealed envelopes with allocation details and a log in code. | Low risk of bias: assessors were blinded at baseline and 12 weeks. Participants were asked not to inform assessors of their group allocation. | Low risk of bias: Loss to follow-up was different across arms - the control group were more likely to attend then the basic group 84.2% follow-up. Used ITT analysis using BOCF. | Low risk of bias: published protocol and reported outcomes. | Low risk of bias weight was measured. |
| Fujimoto et al [[29](#_ENREF_29)] | Low risk of bias: randomisation table. | Unclear risk of bias: allocation concealment not stated. | Unclear risk of bias: not stated. | High risk of bias:  Difference in follow-up rates 87% in the IG provided follow-up data at two years and 65% in the CG. At the end of therapeutic interviews the follow-up rates are not clear. | High risk of bias: analysed groups by those who had follow-up data at 24 months. Did not randomise males therefore male data is not included. | Unclear risk of bias: weight measures not stated but probable that weight was measured at the hospital visits. |
| Gokee La Rose et al [[40](#_ENREF_40)] | Unclear risk of bias: “Participants were randomised to one of two groups.” | Unclear risk of bias – allocation concealment not stated. | Unclear risk of bias: no blinding of outcome was stated. | Low risk of bias: 93% follow-up, similar rates for both groups. No method of imputation. | Unclear risk of bias | Low risk of bias: weight measured. |
| Haapala et al[[35](#_ENREF_35)] | Unclear risk of bias: “Randomisation was performed within gender to one of two groups.” | Low risk of bias: the study nurse was blinded to the randomisation procedure. | Unclear risk of bias: no blinding of outcome was stated. | Low risk of bias: Similar dropout rates for both groups, however the IG dropouts had lost significantly less weight by 3 months than those who continued. Used BOCF or LOCF whichever was higher. | Unclear risk of bias | Low risk of bias: weight measured. |
| Heckerman et al [[22](#_ENREF_22)] | Unclear risk of bias: “Subjects were randomly assigned to 1 of 2 conditions.” | Unclear risk of bias: allocation concealment not stated. | Unclear risk of bias: no blinding of outcome was stated. | High risk of bias: follow-up rates were very low at 6 months, 42% IG and 18% CG. No imputation of missing weights. | High risk of bias: standard deviations not reported | Low risk of bias: weight measured. |
| Imai et al [[17](#_ENREF_17)] | Unclear risk of bias: “Stratification randomisation that considered gender, age and weight.” | Unclear risk of bias: allocation concealment not stated. | Unclear risk of bias: no method of blinding recorded. | Low risk of bias: 92% completed follow-up and dropouts were similar within each group. No method of imputation. | Unclear risk of bias | Low risk of bias: weight measured. |
| Joachim et al[[24](#_ENREF_24)] | Unclear risk of bias: “subjects were randomly allocated to one of four groups.” | Unclear risk of bias: allocation concealment was not stated. | Unclear risk of bias: no method of blinding recorded. | Unclear risk of bias: Follow-up rates were not stated | High risk of bias: standard deviations not reported | Low risk of bias: weight measured. |
| Lally et al[[42](#_ENREF_42)] | Unclear of bias: participants were offered one of three week days and these days were randomly allocated to the three groups by an independent researcher. | Unclear risk of bias: allocation concealment was not stated. | Unclear risk of bias: no method of blinding recorded. | Low risk of bias:86% provided follow-up data. Follow-up rates differed slightly 94% in control, 78% IG 1 and 83% IG 2. | Unclear risk of bias | Low risk of bias: weight measured. |
| Leermakers et al [[41](#_ENREF_41)] | Unclear risk of bias: “Participants were randomly assigned to one of two conditions.” | Unclear risk of bias: allocation concealment not stated. | Unclear risk of bias: no method of blinding recorded. | Low risk of bias: 69% provided follow-up data and attrition did not vary by group. Used BOCF. | Unclear risk of bias | Low risk of bias: weight measured. |
| Linde et al[[30](#_ENREF_30)] | Low risk of bias: random number table. | High risk of bias: no allocation concealment | High risk of bias: study staff were not blinded to participant treatment assignment | High risk of bias: Different follow-ups at 6 months; 64.7% of IG and 76% of CG. | Unclear risk of bias | Low risk of bias: weight measured. |
| Linde et al[[50](#_ENREF_50)] | Low risk of bias: Blocked randomisation at the worksite level (block size 2) computer generated algorithms. | High risk of bias: No allocation concealment | Low risk of bias: no blinding of outcome measure but the measurement team did not participate in intervention delivery. | Low risk of bias: 80.4% provided follow-up data. No method of imputation used for missing weight data. | Unclear risk of bias | Low risk of bias: weight measured at follow-up. |
| Ma et al [[32](#_ENREF_32)] | Low risk of bias: used a covariate-adaptive Efron biased coin method. | High risk of bias: no allocation concealment. | Low risk of blinding: blinding of outcome measures, outcome adjudication and data analysis. | Low risk of bias: 80.5% provided follow-up data at 15 months. Used maximum-likelihood estimation via mixed modelling. | Low risk of bias: analyses conducted according to protocol. | Low risk of bias: weight measured |
| Madigan et al [[27](#_ENREF_27)] | Low risk of bias: table random block sizes of between 2 and 8. | Low risk: opaque sealed envelopes. Participants were blinded to group allocation. | Low risk of bias: weight at three months was collected by independent researchers. | Low risk of bias: 92.4% of IG and 85.7% of CG provided follow-up data. Used BOCF | Low risk of bias: analyses conducted according to protocol. | Low risk of bias: weight measured. |
| Mahoney et al [[23](#_ENREF_23)] | Unclear risk of bias: “The subjects were ranked according to degree of obesity and randomly assigned to groups.” | Unclear risk of bias: allocation concealment not stated. | Unclear risk of bias: no method of blinding recorded. | High risk of bias: 45% provided follow-up data. No imputation | High risk of bias: standard deviations not reported | Low risk of bias: weight measured. |
| Mehring et al [[26](#_ENREF_26)] | Low risk of bias: sequence randomisation was provided by a methodologist not who did not participate in the execution of the study via the programme randomizer. | Low risk of bias: opaque sealed envelopes. | High risk of bias: no method of blinding as GP’s were randomised not individual participants. | High risk of bias: follow up rates differed at three months but did use BOCF. | Unclear risk of bias | Low risk of bias: weight measured. |
| Pacanowski & Levitsky [[13](#_ENREF_13)] | Unclear risk of bias: “Individuals randomised to one of two groups.” | Unclear risk of bias: allocation concealment not stated. | Unclear risk of bias: no method of blinding recorded | Low risk of bias: 83.3% provided follow-up data. Used LOCF. | Unclear risk of bias | Low risk of bias: weight measured |
| Steinberg et al[[36](#_ENREF_36)] | Unclear risk of bias: “Participants were randomised to one of two treatment groups.” | High risk of bias: no allocation concealment | Unclear risk of bias: no method of blinding recorded. | Low risk of bias: follow-up rates of 96% in IG and 95% in CG. Random effects imputation used. | Unclear risk of bias | Low risk of bias: weight measured. |
| VanWormer et al[[20](#_ENREF_20)] | Low risk of bias; Used a computer generated block randomisation list. | High risk of bias: No allocation concealment | Unclear risk of bias: no method of blinding recorded. | Low risk of bias: 87% of IG and 84% of CG provided follow-up data. Used LOCF and added 1.2lbs per follow-up time period. | Unclear risk of bias | Low risk of bias: weight measured. |
| Wing et al [[37](#_ENREF_37)] | Unclear risk of bias: Randomly assigned with their team mates to 1 of 2 groups. A 2:1 randomisation ratio was used. | Unclear risk of bias: allocation concealment not stated. | Low risk of bias: assessors were blinded to treatment allocation | Low risk of bias: 87.5% provided follow-up data and no significant difference between groups. Used BOCF. | Unclear risk of bias | Low risk of bias: weight measured. |

CG= control group IG= intervention group
