# Supplementary material for: Is self-weighing an effective tool for weight loss: a systematic literature review and meta-analysis
Source: Int J Behav Nutr Phys Act. 2015 Aug 21;12:104. doi: 10.1186/s12966-015-0267-4 (PMC4546162; doi:10.1186/s12966-015-0267-4)
Supplement: Additional file 3: — Characteristics of included trials. (DOCX 46 kb) [file 12966_2015_267_MOESM3_ESM.docx]

**Online Additional file 3: Characteristics of included trials**

| Study, Country, design | Inclusion criteria | Participants and % female | Setting | Duration of intervention and follow-up | Intervention | Control/ comparator group |
| --- | --- | --- | --- | --- | --- | --- |
| Allen et al (2013) USA, RCT[[39](#_ENREF_39)] | Aged 21-65 years, BMI 28 -42 kg/m^2^ who had an i phone or Android phone. | n=68  77.9% female | Counselling sessions and smart phone applications but setting not clearly stated. | 6 month intervention and follow-up. | Goals of 150 minutes of MVPA and to lose 5% of weight. 15 healthy eating and PA counselling from a nutritionist coach. Smart phone involved entering calories and physical activity and encouraged to weigh weekly. | The same intervention without the self-monitoring phone application. |
| Anderson et al (2014) UK, RCT[[32](#_ENREF_32)] | Aged 50-74 years who had undergone a polypectomy for adenoma, BMI >25 kg/m^2^ and able to undertake physical activity. | n=329 26% female | Group based sessions, not specified where but recruited through cancer screening programme. | 12 month intervention and follow-up. | 12 month BeWel programme. Three one to one visits in the first three months followed by 9 monthly 15 minute telephone calls. Motivational interviewing, set target to lose 7% body weight, personalised energy prescription of reduction by 600kcal. Given body weight scales for weekly self-monitoring. | Usual care |
| Appel et al (2011) USA, RCT [[26](#_ENREF_26)] | Aged ≥21 years. One or more CVD risk factors. A patient at the practice. Regular access to a computer and basic computer skills. Obese adults not lost ≥5% body weight. | n=415  63.6% female | Primary care, online programme and either remote support or in person support. | 24 month intervention and follow-up | Instructed to weigh weekly then daily. Remote group had 33 phone calls and in person group had 30 group sessions. Based on SCT with motivational interviewing, weight related goals, self-monitoring; weight, exercise and reduced calorie intake. Received feedback on progress. | Received brochures and list of recommended websites promoting weight loss. Met with a weight coach at baseline. |
| Bacon et al (2002) USA, RCT[[40](#_ENREF_40)] | Caucasian, female, 30-45, BMI ≥30 kg/m^2^, non smoker, not pregnant or intending to get pregnant, not lactating, restraint scale >15, practising birth control, premenopausal. | n=78  100% female | Not recorded | 24 weeks intervention followed by 6 month optional maintenance intervention. Follow-ups at 12, 24 and 52 weeks. | Monitor weight weekly and complete food diaries. 24 weekly sessions, 90 minutes in length delivered by dietician. Eating behaviours, nutrition, social support and exercise. Focus on self-monitoring, stimulus control, reinforcement and cognitive change. Monthly group support sessions for weight maintenance. | Intervention about body acceptance, eating behaviour, activity, nutrition and social support delivered by counsellor. Monthly group support sessions for weight maintenance. |
| Batra et al (2013) USA, Cluster RCT[[35](#_ENREF_35)] | BMI ≥25 kg/m^2^ aged ≥21 and written clearance for participation in the study by their physician and absence of significant comorbidities. | n= 4 worksites and 118 employees 76% female | Worksite | 6 month intervention and follow-up | Weekly group sessions for 15 weeks and then semi-monthly. Educational component and forum for discussion and social support. macronutriemt targets. Craving control and weighing daily and self-monitoring food intake as needed. | Delayed intervention. |
| Bertz et al (2012), Sweden, RCT[[34](#_ENREF_34)] | Female, self-reported pre pregnancy BMI 25-35 kg/m^2^, non-smoking, singleton term delivery, intention to breastfeed for 6 months, providing <20% of energy intake as complimentary foods, birth weight of infant >2500g, no illness in mother or infant. | n=68 100% female | Home | 12 week weight loss programme and follow up with a further follow-up at 12 months. | Two groups 1. Diet plan to reduce calories by 500 kcals/day. Received scales to record weight 3 times per week. Two individual behavioural counselling session, bi weekly- text messages to report body weight.  2. All of the above but also encouraged to increase exercise. Given exercise plan and heart rate monitor and activity diary. When they reported their weight they reported number of walks too. | Usual care group |
| Collins (2012) Australia, RCT[[29](#_ENREF_29)] | Access to computer with email/ internet services, pass a health screen test, aged 18 to 60 years, BMI 25-40kg/m^2^, not participating in another weight loss programme. | n=309  58.2% female | Online programme | 3 months intervention and follow-up. | Based on SCT and involved self-efficacy, goal setting and self-monitoring of body weight at least weekly. Individual daily calorie targets for a weight loss of 0.5 kg to 1 kg per week. Completed food and exercise diaries, menu plans and received weekly email newsletters. Enhanced intervention got above and received enrolment reports, personalized e feedback and escalating reminders. | Delayed control group. |
| Fujimoto et al (2002) Japan RCT [[30](#_ENREF_30)] | No previous dietary intervention | n=72  100% female | Hospital outpatient clinics | Intervention, end at approximately 7.2 months. Follow-up 2 years after intervention. | Same as comparator group but told to weigh four times per day during the programme and then daily. Had follow-ups at the hospital every 6 months. | Individual weekly therapy interventions and same follow-ups at the hospital every 6 months. |
| Gokee La Rose (2009) USA, RCT [[41](#_ENREF_41)] | Aged 21-35 years, BMI 27-40 kg/m^2^, no history of eating disorder or substance abuse. | n= 40  % females not reported. | University, group meetings | 10 weeks intervention and follow-up. | Received the same intervention as the comparator group but given digital scales and instructed to weigh daily. Taught how to use the scale much like a blood glucose monitor. Based on weekly weight; a colour scale based on the stop regain model was used to determine whether they needed to modify their behaviour. If in the green zone they were given a small gift. | Diet and exercise goals, behaviour modification skills for both groups over 10 weeks. Weighed at the group weekly meetings and told not to self-weigh at home. |
| Haapalal (2009) RCT location not recorded [[36](#_ENREF_36)] | Aged 25-44 years, BMI 25-36 kg/m^2^, access to mobile phone and internet connection | n=125  79% females | Internet/mobile phone intervention. | 12 months intervention and follow-up. | Encouraged to increase daily physical activity and daily weight reporting via text or website. Dietary records and graphs tracking ones weight. Given mobile phone programme and calculated daily energy requirement. Received feedback via text message whether they achieved their goals. | No intervention |
| Heckerman et al (1978) RCT, USA [[23](#_ENREF_23)] | Must be 15 pounds overweight. | n=23  87% female | Weekly group meetings. | 4 weeks intervention (mid programme), 10 weeks and 6 months follow-up. | Same intervention as comparator/ control but instructed to weigh often between meetings. Stimulus control, self-monitoring, nutritional management, self-management and exercise. | Weighed at 10 weekly meetings and then monthly meetings during 6 months. Told to avoid weighing at home. |
| Imai et al (2008) Japan, RCT [[18](#_ENREF_18)] | Aged 40-70 years, impaired glucose tolerance haemoglobin Alc (HbA1c) levels> 5.5- 6.1. | n=100  83% female | Group sessions and individual sessions. Setting not recorded. | 6 month intervention and follow-up | Self-weighing twice a day: morning and evening. Used diaries to record weight. Educators, registered dieticians and trained nurses delivered the interventions. Portion control, exercise, set dietary goals. Educators gave support, encouragement and feedback. Set a goal of 5% body weight loss. | Received lectures about diabetes, reducing weight and increasing physical activity. Understanding food labelling. Not participant orientated and educators not trained in group dynamics. |
| Joachim et al (1975) USA, RCT [[25](#_ENREF_25)] | Resident at location and no known physiological case of weight. | n= 32  % female not reported | Institution for mildly retarded adults. | 8 weeks intervention and follow-up. Additional follow-up at 16 weeks. | 3 IG’s- 1. Weigh and record twice per day. Instructed to lose weight by any means. 2. Weigh and record twice per day. 3. Instructed to lose weight by any means. Half of each group received weekly contact and the remainder no contact. | No intervention |
| Lally et al (2008) UK, RCT[[43](#_ENREF_43)] | Aged ≥ 18 years old and BMI ≥25 kg/m^2^ | n=104 66% female | Managers of local businesses sent an email to employees to contact the research team. | 8 weeks intervention and follow-up. | 2 IG’s – Both groups given a leaflet that promoted 10 top tips, 7 were estimated to create a daily deficit of 800-900 kcals and three were for energy expenditure. Were encouraged to weigh themselves regularly and given a self-monitoring form where they could record their weight daily. IG 1. Were weighed on a weekly basis and IG 2. Were weighed on a monthly basis. | Delayed control group. |
| Leermakers et al (1998) USA, RCT[[42](#_ENREF_42)] | Aged ≥ 18 years old, delivered a baby in the last 3 to 12 months and exceeded pre pregnancy weight by at least 6.8 kg. BMI ≥22 kg/m^2^. | n=90  100% females | Correspondence and regular phone calls. | 6 month intervention and follow-up | Regular phone calls and weekly weight reporting. Aerobic exercise program of walking. 16 Weekly, bi weekly then monthly written lessons covering nutrition, exercise, behaviour change strategies, set goals of 1000-1500 kcals/day, fat less than 20%. Problem solving within groups. | Given a leaflet and information brochure about healthy eating and exercise. |
| Linde et al (2011) USA, RCT [[31](#_ENREF_31)] | 18 to 65 years, BMI 25-35 kg/m^2,^ not diabetic, not trying to lose weight, not pregnant or has been within the last year. University employees. | n=68  72.7% female | University and home | Single session intervention and 6 months follow-up | Given single intervention session and a self-help treatment manual, weighing scales, pedometer, food composition and physical activity book. Completed 24 weekly self-monitoring records of weight per day, frequency of weighing, pedometer use and dietary activities. Set goals and signed written contracts. | Single page flyer about nutrition |
| Ma et al (2013) USA, RCT[[33](#_ENREF_33)] | ≥18 years, BMI ≥25 kg/m^2^, presence of pre diabetes mellitus or metabolic syndrome. | n=241  46.5% female | Primary care | 15 month intervention and follow-up | 3 month intensive intervention followed by 12 months maintenance phase.   1. Coach led face to face group meeting 2. Self-directed DVD intervention   Both groups received 12 sessions based on Diabetes Prevention Programme. Access to website to monitor weight and physical activity. Given a pedometer and scale. Received standard bi-weekly reminders to self-monitor and monthly motivational messages during maintenance phase. Coach led group also had weekly weigh ins, food tasting, physical activity sessions, individual action plans. | Usual care from their primary care provider |
| Madigan et al (2014) UK, RCT[[28](#_ENREF_28)] | BMI ≥30 kg/m^2^, aged ≥18years and not self-weighing once per week or more. | n=183  63% female | Primary care | 3 month intervention and follow-up. | Given a set of scales and instructed to weigh daily and record it on the card provided. Sent weekly text message reminders. Two individual weight management consultations about general strategies to aid weight loss and set a target of losing 0.5 kg per week. | Same weight management consultation about general strategies to aid weight loss |
| Mahoney et al (1973) USA, RCT [[24](#_ENREF_24)] | Minimum age 17 years, not pregnant, physicians consent, minimum of 10% overweight. Recruited by newspaper advert. | n=53  90.5%females | Not recorded | 4 week intervention and follow-up at 4 weeks and 4 months. | Instructed to weigh bi-weekly for 7 weigh ins and keep daily weight graph. Given a behavioural diary to record “fat thoughts”, “thin thoughts”, instances of indulgence and instances of restraint. Four IG: self-reward, self-punishment, both, no reward or punishment. | Received stimulus control booklets |
| Mehring et al (2013) Germany, Cluster RCT[[27](#_ENREF_27)] | BMI ≥25kg/m^2,^ ≥18years, recruited through primary care. | n=186  68.8% female | Primary care and web-based programme. | Three months intervention and follow-up. | Web-based coaching programme after receiving referral from physician (12 sessions). Recommendations by physician were uploaded to the website and individual coaching plan developed. Received daily text messages, three telephone calls from GP and to self-monitor diet and PA. Diary entries for weight and waist circumference. | Usual care from GP |
| Pacanowski et al (2011) USA, RCT[[44](#_ENREF_44)] | BMI ≥27kg/m^2^, aged ≥18years , not diabetic and did not have history of eating disorder. | n=162  81.9% females | 12 month internet intervention | Follow ups at six months and 12 months after the initial session. | Received an initial consultation and then an internet programme that provided daily feedback of individual’s daily weight trends via a graph. After 8 entries a green line appears 1% below current weight. The aim was to lose 10% of weight in one year. Were contacted if they did not submit weights. | Delayed control group |
| Steinberg et al (2013) USA, RCT [[37](#_ENREF_37)] | 18 to 60 years, BMI 25 to 40kg/m^2^, maximum weight 330lbs. Access to internet. | n=91  75% females | One group session followed by emails and internet. | 6 months intervention and follow-up. | Given set of scales and told to weigh daily. Received weekly tailored feedback via email and a web based graph of weight trends over time. Set targets of calories per day and minutes of MVPA. 22 weekly lessons on behavioural control and aim -0.5 lbs per week. Placed in categories of self-weighing frequency each week and given reinforcement of specific strategies for adopting daily weighing. | Delayed intervention |
| Van Wormer et al (2009) USA RCT [[21](#_ENREF_21)] | Employees of health partners aged over 18 years, BMI ≥32 kg/m^2^. Willing to perform daily weighing. Invited through worksite. | n=100  91% female | 10 counselling calls and telemonitoring | 6 months intervention and follow-up. | Given a set of scales that beamed weight data to researchers who could proactively follow-up those who gained weight. The scales prompted participants to answer questions about physical activity and diet behaviours. Instructed to complete a weekly weight chart. Given programme manual: reduce calorie deficit by 500 kcals, 10,000 steps/ day, stress management, cognitive reframing, problem solving/ relapse prevention. Given a pedometer, food and activity logs. | Delayed intervention. |
| Wing et al (2010) USA , RCT (study 2) [[38](#_ENREF_38)] | Able to use the website and complete a food diary. Be older than 18 years and BMI greater than 25 kg/m^2^. | n=128  % female not stated | Online programme using a website and GP sessions. | 12 weeks intervention and follow-up. | An enhanced version of the Shape up RI programme (online weight loss programme), participant’s submitted data every two weeks. Self-monitor weight, diet and physical activity daily and received weekly feedback that was automated. One group session: introduction about energy balance and importance of self-monitoring. | Received standard ‘shape it up’ programme. |

BMI = body mass index, BOCF = baseline weight observed carried forwards CG= control group, IG= intervention group, LOCF = last weight observed carried forwards, MVPA = moderate to vigorous physical activity, SCT= social cognitive theory
