# Supplementary material for: Is self-weighing an effective tool for weight loss: a systematic literature review and meta-analysis
Source: Int J Behav Nutr Phys Act. 2015 Aug 21;12:104. doi: 10.1186/s12966-015-0267-4 (PMC4546162; doi:10.1186/s12966-015-0267-4)
Supplement: Additional file 4: — Number of behaviour change techniques used in each study’s intervention by cluster group. (DOC 21 kb) [file 12966_2015_267_MOESM4_ESM.doc]

Online Additional file 4: Number of behaviour change techniques used in each study’s intervention by cluster group

	
Allen	
Anderson	
Appel	
Appel web	
Bacon	
Batra	
Bertz	
Collins	
Fujimoto	
Gokee laRose	
Haapalal	
Heckerman	
Imai	
Joachim 1	
Joachim 2	
Lally	
Leermakers	
Linde	
Ma	
Madigan	
Mahoney SR	
Mahoney SP	
Mahoney SR	
Mahoney SM	
Mehring et al	
Pacanowski	
Steinberg	
VanWormer	
Wing	Total number of techniques	
*Percentage of use of possible	
Goals and planning (9)	5	7	7	7	0	5	6	5	3	4	2	0	8	2	1	5	3	4	7	4	5	5	5	5	6	2	3	6	5	12
7	48.
6	
Reward and threat (5)	0	0	0	0	0	0	0	1	0	1	0	0	2	0	0	0	0	3	0	1	2	0	2	0	0	1	0	0	0	13	11.
8	
Regulation (2)	0	0	0	0	0	0	0	0	0	0	0	0	0	0	0	0	0	0	0	0	0	0	0	0	0	0	0	1	0	1	2.3	
Antecedents (1)	0	0	1	1	0	0	0	0	0	0	0	0	0	0	0	0	0	0	0	1	0	0	0	0	0	0	0	0	0	3	13.
6	
Identity(1)	0	0	0	0	0	0	0	0	0	0	0	0	0	0	0	0	0	0	0	0	0	0	0	0	0	0	0	0	0	0	0.0	
Self-belief (2)	0	0	0	0	0	0	0	0	0	0	0	0	0	0	0	0	0	0	0	0	1	1	1	1	0	0	0	0	0	4	9.1	
Covert learning (1)	0	0	0	0	0	0	0	0	0	0	0	0	0	0	0	0	0	0	0	0	0	0	0	0	0	0	0	0	0	0	0.0	
Feedback and monitoring (4)+	3	3	4	4	2	2	2	3	4	2	1	1	1	2	2	3	4	0	3	2	4	4	4	4	3	1	3	4	4	63	54.
3	
Social support (3)	2	1	2	2	1	1	1	1	0	0	0	0	0	0	0	0	0	0	1	0	0	0	0	0	1	0	0	0	1	7	10.
6	
Shaping knowledge (1)	1	1	1	1	0	1	0	0	1	0	0	0	1	0	0	0	1	1	1	0	1	1	1	1	1	0	1	1	0	12	54.
5	
Natural consequences
(3)	0	0	1	1	1	0	0	0	0	1	1	0	2	0	0	0	0	0	0	1	0	0	0	0	1	1	0	0	1	10	15.
2	
Comparison of behaviour (4)	0	1	0	0	1	0	0	2	0	1	0	0	0	0	0	0	0	1	1	0	2	2	2	2	0	0	0	0	1	14	15.
9	
Associations (1)	0	0	1	1	0	0	0	0	0	0	0	0	0	0	0	0	0	0	0	0	0	0	0	0	0	0	0	0	0	2	9.1	
Repetition and substitution (3)	0	0	0	0	0	0	1	0	0	0	0	0	0	0	0	0	1	0	0	0	0	0	0	0	0	0	1	0	0	2	3.0	
Use of follow up prompts (1)	1	1	1	1	0	1	0	1	0	0	0	0	0	0	0	0	0	0	0	1	0	0	0	0	1	0	0	1	0	5	22.
7	
Total	1
2	1
4	1
8	1
8	5	1
0	1
0	1
3	8	9	4	1	1
4	4	3	8	9	9	1
3	1
0	1
5	1
3	1
5	1
3	1
3	5	8	1
3	1
2	29
9		
1.   Joachim weigh and record and instruction 2. Joachim weigh and record	*Percentage of techniques used out of all those within the
domain.	+Added monitoring by others into this category.
